# Using Modified Equipment in Field Hockey Leads to Positive Transfer of Learning Effect

**DOI:** 10.3389/fpsyg.2021.653004

**Published:** 2021-04-29

**Authors:** Johanna E. A. Brocken, John van der Kamp, Matthieu Lenior, Geert J. P. Savelsbergh

**Affiliations:** ^1^Amsterdam Movement Sciences, Faculty of Behavioral and Movement Sciences, Vrije Universiteit Amsterdam, Amsterdam, Netherlands; ^2^Research Centre for Exercise, School and Sport, Windesheim University of Applied Sciences, Zwolle, Netherlands; ^3^Department of Movement and Sport Sciences, Faculty of Medicine and Health Sciences, Ghent University, Ghent, Belgium; ^4^Faculty of Sports and Nutrition, Amsterdam University of Applied Sciences, Amsterdam, Netherlands

**Keywords:** cross-education, positive transfer, field hockey, motor learning, dynamical systems theory, modified equipment, transfer of learning

## Abstract

Cross-education is the phenomenon in which repeated practice of a unilateral motor task does not only result in performance improvement of the trained limb, but also in the untrained contralateral limb. The aim of this study was to test whether cross-education or positive transfer of learning is also achieved for tasks in which both limbs contribute in different ways by using modified equipment that switches the limbs’ role. To this end, a reverse field hockey stick was used that requires a mirroring of arm and hand use and dominance (i.e., right hand on top of the hockey stick instead of the left hand). Two groups of young skilled female field hockey players participated in a crossover-design, in which participants received four training sessions with a reverse hockey stick followed by four training sessions with a regular hockey stick, or vice versa. In a pre-test, intermediate test (following the first intervention period), a post-test (after the second intervention period) and a retention test, participants’ performance on a field hockey skill test with a regular hockey stick was measured. The results revealed that training with the reversed hockey stick led to significantly increased improvements compared to training with a regular hockey stick. We conclude that modified equipment can be used to exploit positive transfer of learning by switching the limbs’ roles. The findings are discussed by referring to the symmetry preservation principle in dynamic systems theory and have clear practical relevance for field hockey trainers and players seeking to further improve field hockey skills.

## Introduction

In the late 19th century, Scripture dubbed the term “cross-education” to refer to the intriguing training effect that he had witnessed in his co-authors Brown and Smith (Scripture et al., 1894). Miss Brown performed strength training by squeezing a rubber bulb, while Miss Smith practiced to sting a needle through holes that became increasingly smaller; each subsequent hole was smaller than the previous one. Crucially, both co-authors performed the training with a single limb; yet, the training effects were not restricted to the trained limb, but also included improved performance of the untrained contralateral limb. This interlimb transfer effect was referred to as cross-education (Barss et al., 2016). Recently, this cross-education or interlimb transfer has also been reported for complex sport activities. For example, Haaland and Hoff (2003) demonstrated in a group of experienced adult soccer players that practicing soccer tasks such as dribbling, passing, and kicking with one leg, significantly improved performance in both legs, including the untrained leg. Similar interlimb transfer was reported in children for throwing and dribbling in basketball (Stöckel et al., 2011; Stöckel and Weigelt, 2012). Surprisingly perhaps, the interlimb transfer did not only take place from the dominant to the non-dominant limb, but also from the non-dominant to the dominant limb; in fact, the latter effect was found to be stronger.

There is no clear consensus on the mechanism underlying this cross-education effect or positive transfer of learning. Nevertheless, most explanations hold that, since the two limbs are seen as independent systems, any transfer of the *intra*limb changes from one limb to the other, must involve a spill over to and from an overarching (specialized) representation that controls both limb systems. For example, Stöckel and Weigelt (2012) argue that lateralized representations exist that determine different aspects of movement control (e.g., sequencing, visuospatial control, and force regulation). These representations are thus specialized in controlling different aspects in both the contralateral and ipsilateral (via the corpus callosum) limbs (Sainburg, 2002), and are believed to underpin interlimb transfer or cross education. Further to this point, because the representations are lateralized, interlimb transfer strength can be asymmetric dependent on the movement aspect involved (Stöckel and Weigelt, 2012). Presumably, sequencing is an aspect of the movement that is left-hemispheric lateralized. Hence, initial practice with the right limb results in increased transfer to the untrained limb as compared to practice with the left limb. Conversely, visuospatial control is right-hemispheric lateralized, and hence, practice with the left limb results in greater transfer -also in right-handers.

In this article, we take an ecological dynamics approach to motor skill learning (Davids et al., 2013; see also Davids et al., 2008). Ecological dynamics combines ecological psychology (Gibson, 1979) and dynamical systems approaches (Kugler and Turvey, 1987; Kelso, 1995) to explain how at the level of the performer-environment relationship functional behaviors transpire. In ecological dynamics, performers are considered as complex neurobiological systems (i.e., composed of many interacting components) that self-organize under constraint of task and environment (Newell, 1986). Hence, rather than being prescribed by neural representations, movement coordination patterns spontaneously emerge from the interactions between system components and the specific performance context (Davids et al., 2013). It is pertinent from the ecological dynamics approach that in complex sport activities such as kicking, throwing, dribbling, long jumping, and so on, movement coordination patterns rarely involve a single limb in complete isolation from or independent of the other limb and the specific performance context. Typically, the second (often the non-dominant) limb supports the first (dominant) limb. For instance, in kicking a ball, the swinging leg is indeed critical in propelling and directing the ball. Yet, as skilled goalkeepers are aware of, also the orientation of the supporting leg, which is placed next to the ball, is usually aligned with the direction of the kick (Savelsbergh et al., 2002). In other words, within complex sports activities the limbs are more adequately considered as a softly assembled system in which one limb is leading and the other limb is supporting (or enslaved), but always move in close coordination and constrained by the performance context. Consequently, the interlimb transfer referred to in cross-education then would involve a switching of roles, with the more abstract coordination (or phasing) between limbs and the performance context being preserved. From that point of view, the intralimb representation argument as for instance used by Stöckel and Weigelt (2012), cannot fully account for the effect of cross-education.

In fact, in a now classic series of studies on movement coordination based on the dynamic systems approach, spontaneous transfer of interlimb coordination patterns with practice has been demonstrated more often (Zanone and Kelso, 1992, 1997; Kelso, 1995; Kelso and Zanone, 2002; Magne and Kelso, 2008). Due to the dynamic interactions among its components, a change in one component affects the whole movement system. Accordingly, learning involves the relatively permanent alteration of the *entire* landscape of movement coordination patterns, thus transcending the specific pattern that is practiced. This may involve negative transfer of learning, where pre-existing untrained coordination patterns become less stable or disappear, or positive transfer of learning, where untrained coordination patterns spontaneously emerge or stabilize. Zanone and Kelso (1997), for example, had participants practice rhythmic hand movements in a novel phasing imposed by a metronome, in which one hand led the other with a 90° relative phase. This resulted not only in the novel 90° pattern becoming stable, but also the spontaneous emergence of the 270° pattern. The 270° relative phase is the symmetry partner of the 90° relative phase; that is, it is the same phasing but with the leading-lagging roles of the hands switched. Similarly, the reverse positive transfer occurred to the untrained 90° after practicing the 270° relative phase. Zanone and Kelso (1997) argue that these spontaneous transfers indicate that any alterations of the landscape of movement coordination patterns constituted by two limbs in specific performance context are constrained such that they preserve symmetry. Symmetry means that properties of a system (here: the coordination pattern) remain unchanged after a transformation of the system’s components (here: the limbs) (Jeka and Kelso, 1995, p. 361). The preservation of symmetry allows the limbs to shift roles without changing the underpinning dynamics of coordination; the limbs are functionally equivalent (Kelso and Zanone, 2002).

The ecological dynamics approach holds that practitioners can facilitate learning of complex sport activities by (re-)arranging the performance or practice context (Woods et al., 2020). The resultant change in constraint, encourages the performer to actively explore new or more stable movement coordination patterns. Modifying sporting equipment is a key method for rearranging the practice context in order to induce a performer’s search for functional movement patterns (Chow et al., 2015; Renshaw and Chow, 2019). Indeed, studies using modified sporting equipment show that changing the equipment can have a positive effect on performance and learning. For example, researchers have shown positive effects of modified racket size or net height in tennis (Buszard et al., 2014, 2016; Timmerman et al., 2015; Limpens et al., 2018), or modifying a the mass distribution of the ball in field hockey (Brocken et al., 2020). These beneficial effects are typically attributed to implicit shaping and/or increasing the learners adaptability. In this study, we examine if modified equipment can also be utilized to induce interlimb transfer or cross education. Following the principle of preservation of symmetry, it is predicted that transfer or cross education can occur among coordination patterns in which the limbs have reversed roles (i.e., leading and supporting). Hence, practice by switching the leading and supporting roles may especially stabilize the underpinning dynamics of *inter*limb coordination, rather than the individual (intra)limb kinematics. Accordingly, it may be expected that practice with switched roles enhances the habitual or preferred coordination pattern more than practice of the habitual coordination pattern itself.

**FIGURE 1:**
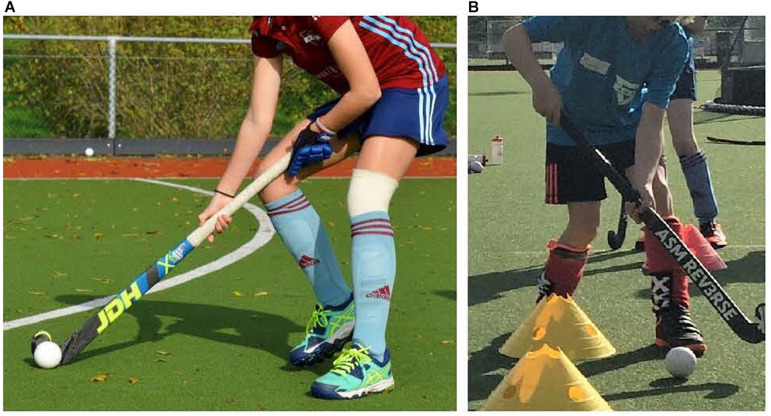
**(A**) The regular stick handled with the left hand on top and the right hand on the lower part (left panel); **(B)** the ASM REV3RSE stick handled with the right hand on top and the left hand on the lower part (right panel).

We test this hypothesis in a complex sport task analogous to those that having been used to study cross education in basketball, soccer and the long jump (see Stöckel et al., 2011; Stöckel and Weigelt, 2012; Focke et al., 2016), but with a more apparent coordination between the limbs. Consequently, we used field hockey, in which players use both hands to handle the stick. Importantly, according to the rules of the game, all players use identically designed sticks that enforce it to be handled with the left hand on the upper part and the right hand on the lower part of the stick. Typically, the left hand is used to hold and rotate the stick (Nederlof, 2018), while the right hand supports the stick and positions the stick relative to the ball (Figure 1A). Therefore, the left hand is often considered as leading/dominant, and the right hand is seen as supporting/non-dominant. To switch roles of the hands, we introduced a modified hockey stick with a mirrored design: the ASM REV3RSE stick^1^, which is used with the right hand holding the upper part and the left hand steering the lower part of the stick. The ASM REV3RSE stick was designed to challenge the adaptability of field hockey players (Figure 1B)^2^.

Following Stöckel et al. (2011), a cross-over design was used to examine practice effects of the regular and modified (or mirrored) stick in group of high skilled youth players. The technical field hockey skills with the regular stick were tested immediately before and after the first and second practice period, and a week after the second practice periods. We expected that practice with both sticks would improve children’s field hockey skills with the regular stick, but, as per cross education, this improvement was predicted to be larger after training with the modified hockey stick, because it is thought to more strongly stabilize the underlying interlimb coordination dynamics. However, it is important to note beforehand that we examined learning by measuring the time the children needed to complete a series of field hockey skills, instead of directly assessing the stability of the movement coordination patterns. We thus presumed that time reflect coordinative skill.

To sum up, we aim to investigate to what extent modified sporting equipment can be used to facilitate learning in field hockey. In particular, we examine whether modified equipment can be utilized to produce interlimb transfer or cross-education. If this indeed is confirmed, then this would have immediate and more long-term effects on sport coaching practice. That is, it should result in the direct application of the ASM REV3RSE stick in field hockey practice, but it should also stimulate search for ways of exploiting the preservation of symmetry principle with and modifying equipment in other for other sports activities.

## Materials and Methods

### Participants

Sixty-eight girls aged between 9.58 and 12.54 years (mean ± SD: 11.37 ± 0.71 years) were recruited from six youth teams of two local Dutch hockey clubs. All six teams trained twice a week for 1.5 h. At the time of the experiment, all teams were playing in the highest level of their regional competition (premier division) of their age group. The Netherlands is divided in eight regional competitions. For this age group, there are only regional and no national competitions. The teams were randomly assigned to group A (i.e., first practice period with the reverse stick and the second period with the regular stick) or group B (i.e., first regular stick and then the reverse stick). *A priori* power analysis for an ANOVA with repeated measures indicated a minimal sample size of 36 participants (α = 0.05, 1-β = 0.95, *f* = 0.25). None of the participants had prior experience with the reverse hockey stick. Guardian consent to participate in the study was provided for all children before the study started. Ethics approval was granted by the Ethical Committee of the Vrije Universiteit (VCWE-2018-031R1).

### Equipment and Apparatus

A regular hockey stick and the ASM REV3RSE hockey stick were used (Figures 1A,B). The reverse stick was provided by the experimenters, while the participants used their personal regular stick. There were two different lengths of the ASM reverse stick, which had a weight of 430 and 520 g. The children used an ASM reverse stick with the same length as their regular stick. The length of the hockey stick depends on the length of the child. The regular hockey sticks of the children were approximately the same weight as the ASM reverse stick (a maximum difference 20 g). The regular stick is rounded at the right face side, while for the reverse stick the left face side is rounded. Since the rules do not allow to contact the ball with the rounded side of the stick face, the two sticks require mirrored coordination pattern. That is, the regular stick requires players to handle it with the left hand on the upper part and the right hand on the lower part of the stick, while the reverse stick requires handling it with the right hand on the upper part and the left hand on the lower part. Notice that the reverse stick is new to the players, and that its use is prohibited in official matches.

**FIGURE 2:**
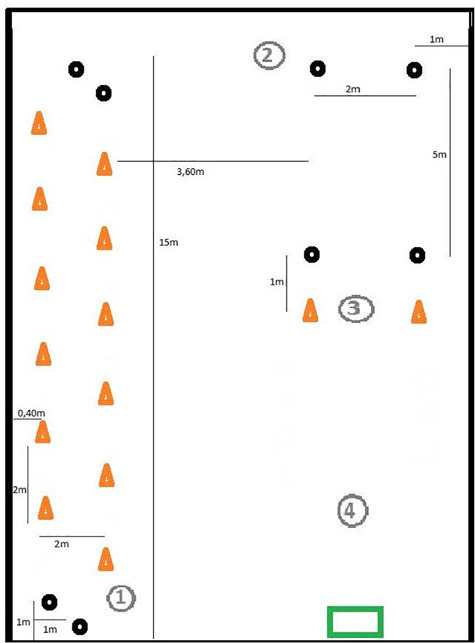
The field hockey skills track. Note that the cones represent cones 35 cm high and the circles represent cones 8 cm high. The rectangle is a 100 cm wide goal. Figure retrieved from Brocken et al. (2020).

A validated field hockey skills test was used that consisted of items assessing ball control and shooting skills (Mekel and Cremer, 2016; Brocken et al., 2020). The test is a track consisting of different skills that need to be performed as quickly as possible in a fixed order (see Figure 2). In the track, the child starts behind the black cones. When the child runs through the black cones, the time recording is initiated. First, a large slalom is performed (1), next the child runs to the second part of the track (2), where they run backwards with the ball. At (3), the child performs a figure eight around the cones. Lastly, the child tries to score a goal at (4). The main measure is the total time needed by the participant to complete the entire track, with the time recording being stopped the moment the ball passed the goal line. In the case the participant missed the goal, she must run across the goal line and then the time was stopped.

### Procedure and Design

**FIGURE 3:**
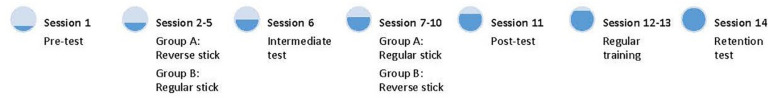
Cross-over design.

A crossover-design with two groups was used (Figure 3). Because the participants in a crossover-design serve as their own controls, this reduces the influence of confounding covariates. In addition, this design was considered fair, since every participant had the opportunity to practice with the reverse hockey stick and profit from its advantages, if any. The participants were divided in two groups, based upon the team they were playing: group A (*N* = 33) used the reverse stick in the first period and their regular hockey stick in the second period (i.e., reverse – regular); and group B (*N* = 35) used their regular hockey stick first and practice with the reverse stick in the second period (i.e., regular – reverse). The reverse stick was used 20 min per training session (explained below); during the remainder of the training, the participants all used their regular hockey stick.

The study extended over a period of 7 weeks (10 practice sessions + 4 test sessions). In session 1, 6, 11, and 14, children’s field hockey skills were assessed in a pre-test, intermediate test, post-test and retention test. To this end, the field hockey skills test was performed with the regular field hockey stick, and during a regular training session. First, a research assistant (a senior hockey player with more than 10 years of experience) demonstrated how to perform the hockey skills test. Subsequently, the participants were allowed to practice the test once. After this, the participants had to perform one test trial. Total time was recorded with a hand-held stopwatch.

In sessions 2–5 (i.e., first practice period) and 7–10 (i.e., second practice period), the interventions took place. In each practice session, both groups performed exactly the same exercises (i.e., lasting approximately 15–20 min), but each group did these with either the regular or reverse hockey stick (i.e., according to the experimental design). The exercises were basic technical exercises, with themes like controlling the ball, running with the ball or passing the ball (see Supplementary Appendix 1, 2). The practice sessions were led by the regular trainer of the team. To avoid “Hawthorne”-effects, the test personnel were not present during practice. Before the practice sessions started, however, the exercises were extensively explained to the trainers, to ensure that both groups performed the exercises similarly. Finally, between the post-test and the retention test (i.e., sessions 12 and 13), participants in both groups received regular training using their regular stick.

### Statistical Analysis

The time (in seconds) that participants needed to perform the field hockey skills test served as the main dependent variable. With this measure, the change in time between subsequent tests (i.e., first practice period between pre- and intermediate tests, the second practice period between the intermediate and post-tests, and the third practice period between the post- and retention tests) was calculated. For analysis, we first assessed differences in time between groups at the pre-test using an independent *t*-test. For *t*-tests, Cohen’s *d* was calculated to determine effect size. Large effect size were *d* > 0.8, medium-sized effects *d* > 0.5, and small effects *d* > 0.2 (Cohen, 1988). Because this analysis revealed initial differences between the two groups, an analysis of covariance (ANCOVA) with repeated measures to compare the changes across practice periods was performed. Pre-test scores were used as a covariate. Accordingly, change in time was submitted to a 2 (group: A and B) × 3 (practice period; first, second, and third) ANCOVA with repeated measures on the last factor. In case the assumption of sphericity was violated, we reported Greenhouse–Geisser corrections. For ANCOVA, effect sizes were determined using partial eta squared ($\eta_{p}^{2}$). Large magnitudes of effects were $\eta_{p}^{2}>0.14$, medium-sized effects $\eta_{p}^{2}>0.06$, and small effects were $\eta_{p}^{2}>0.01$ (Cohen, 1988). *Post hoc* comparisons were performed using *t*-tests with Bonferonni corrections. Finally, one-sample *t*-tests with Bonferonni corrections were performed to examine whether the change in time scores significantly exceeded zero (and thus pointed to a significant improvement). All statistical tests were performed using SPSS 25.0. The level of significance for all tests was set *a priori* to 0.05.

## Results

Thirteen participants were excluded from the analyses because they either did complete less than four sessions in the first and/or second practice period, or did not complete all tests. Of the excluded participants, nine belonged to group A (reverse – regular) and four belonged to group B (regular – reverse). One outlier from group B was removed due to measurement error (i.e., the time in one of the tests was less than 10 s, which is impossible for finishing the track). Consequently, the analyses were performed with 54 participants in total (i.e., 24 participants in group A and 30 participants in group B).

**FIGURE 4:**
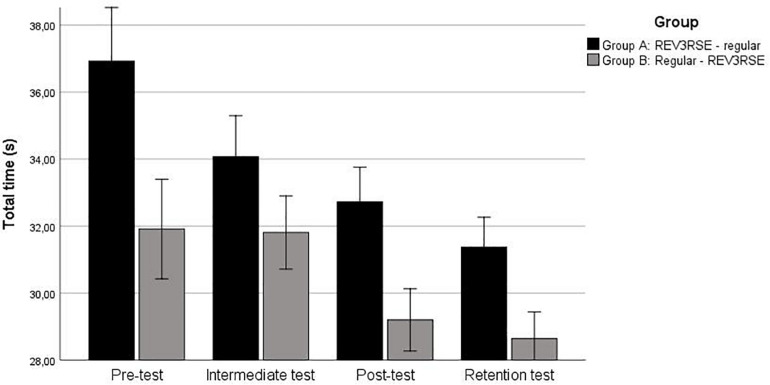
Total time on each subsequent test. Error bars represent 2 SE.

The time (in seconds) that participants needed to perform the field hockey skills track is shown in Figure 4. An independent *t*-test showed that the time at the pre-test for group B was significantly faster (*M* = 31.91, SD = 2.37) than for group A (*M* = 36.93, SD = 5.52), *t*(52) = −4.493, *p* < 0.001, *d* = 1.18. Therefore, the pre-test times were used as covariate in subsequent analyses.

**FIGURE 5:**
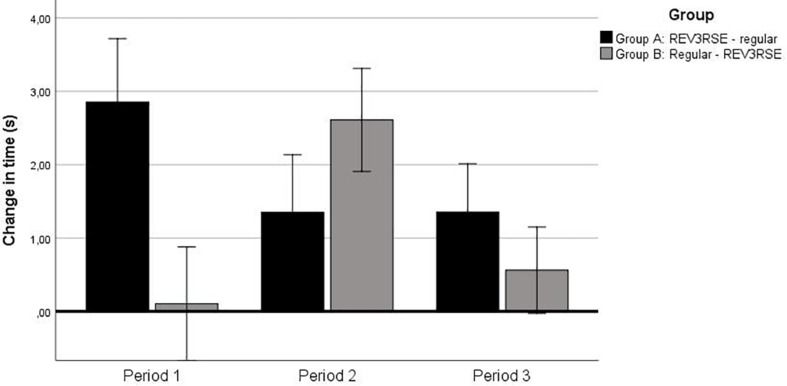
Change in time (improvement) across the three periods. Error bars represent 2 SE. The change in time refers to differences in times between subsequent tests, i.e., (1) pre-test versus intermediate test, (2) intermediate test versus post-test, and (3) post-test and retention test.

Next, a RM-ANCOVA on change in time scores with time in the pre-test as covariate was performed. The improvement (i.e., positive change in time) across the three practice periods is depicted in Figure 5. A significant interaction of the covariate pre-test by practice period was revealed, *F*(1.78,90.79) = 3.36, *p* = 0.04, $\eta_{p}^{2}>0.06$. The latter interaction indicated that longer times on the pre-test were associated with larger change in time scores. However, even after taking account of this covarying variable, a main effect for practice period, *F*(1.78,90.79) = 3.40, *p* = 0.04, $\eta_{p}^{2}>0.06$, and the interaction between practice period and group remained, *F*(1.78,90.79) = 6.72, *p* = 0.003, $\eta_{p}^{2}>0.12$.

*Post hoc* comparisons indicated that for group A the improvement in the first practice period (i.e., with the reverse stick) was significantly larger compared to improvements in the second practice period (i.e., with the regular stick), *t*(23) = 2.61, *p* = 0.016, and the third practice period (i.e., with the regular stick), *t*(23) = 3.13, *p* < 0.001. The improvements in the second and third practice periods (both with the regular stick) did not differ from each other. For group B, the *post hoc* analyses indicated that the improvement in the first practice period (i.e., with the regular stick) was significantly smaller than the improvement in the second practice period (i.e., with the reverse stick), *t*(29) = −4.40, *p* < 0.001, but not with the third practice period (i.e., with a regular stick). Also, the improvement in the second practice period (with the reverse stick) was larger than in the third practice period (with the regular stick), *t*(29) = −1.17, *p* < 0.001.

*Post hoc* analyses further indicated that in the first practice period group A (with reverse stick) improved significantly more than group B (with regular stick), *t*(52) = 4.74, *p* < 0.001. In the second practice period, however, the larger change score for group B (with reverse stick) compared to group A (with regular stick), just failed to reach significance (i.e., *p* = 0.020, with α = 0.017), but showed a large effect size (*d* = 0.66). In the third practice period (i.e., both groups with regular stick), no significant differences in change score were present. Finally, an ANCOVA on the total change score from the pre-test to the retention test with time in the pre-test as covariate was performed to analyze whether the total change in time from the pre-test to the retention test for the two groups differed significantly. A significant interaction of the covariate pre-test by practice period was revealed, *F*(1,51) = 125.9, *p* < 0.001, $\eta_{p}^{2}>0.71$. The latter interaction indicated that longer times on the pre-test were associated with a larger improvement. The ANCOVA also revealed a main effect for group, *F*(1,51) = 4.29, *p* = 0.043, $\eta_{p}^{2}>0.78$, indicating that group A showed the larger improvement across the entire practice period.

The analyses were concluded with a series of one-sample *t*-tests to verify whether the change scores exceeded zero. This revealed that both groups improved test performance after each practice period (*t*’s > 2.57, *p*’s < 0.016), except for group B in the first practice period (with the regular stick).

## Discussion

The aim of this study was to test whether equipment modification can be used to exploit the positive transfer benefits stemming from the preservation of symmetry, which has previously been observed among interlimb coordination patterns (Zanone and Kelso, 1992, 1997). In other words, we used a modified (mirrored) field hockey stick during practice that required handling the stick with the coordination between the two upper limbs switched compared to the handling the regular stick. The leading left arm took the supporting role, while the supporting right arm took the leading role, yet the movement coordination between the leading and supporting arms (rather than left and right arms) remained functionally equivalent. We examined whether practice with the modified stick enhanced performance with the regular (non-modified) stick. The results show that practice with both the regular and the modified hockey stick improved the children’s field hockey skills with the regular hockey stick. Critically, however, the improvements after practice with the reverse hockey stick were more systematic and significantly larger than with the regular hockey stick. Therefore, the use of modified equipment indeed enhanced learning in young, high-skilled field hockey players. We also observed that the total learning, as indicated by the improvement achieved between pre-test and the retention test was larger for the group that started practice with modified stick. A similar sequence effect was reported by Stöckel and Weigelt (2012) in a throwing task. Yet, we are careful interpreting the current observation, because it may also have originated from differences in initial skill level between the groups.

To develop our hypotheses with respect to the modified (mirrored) equipment we used concepts from the ecological dynamics approach, which is grounded in ecological psychology and dynamical systems theory. According to ecological dynamics, coordinated movement behaviors emerge from the dynamic interactions between components of the movement system constrained by the specific performance context (Newell, 1986; see also Davids et al., 2008, 2013). It follows that a change in one component affects the whole movement system. Consequently, learning affects the *entire* landscape of movement coordination patterns, which comprises all the available stable (or preferred) and unstable (non-preferred) movement coordination patterns for an individual learner on a particular task. Since the stability of these coordination patterns are not independent of each other, practicing one movement coordination pattern can affect the entire landscape of coordination patterns. This underpins positive and negative transfer of learning. For example, Zanone and Kelso (1992, 1997) showed that symmetry partners in bimanual coordination patterns (i.e., where different combinations of components of the movement system show similar coordination tendencies, such as when leading and supporting roles of two limbs are interchanged) may be especially conducive for positive transfer (Jeka and Kelso, 1995; see also Kelso and Zanone, 2002). The modified field hockey stick exploits this symmetry preservation principle: it enforces practice of the uncommon mirrored handling of the stick (i.e., with the right arm leading and the left arm following), and in doing so, also spontaneously stabilizes the preferred, dominant handling of the stick (i.e., with the left arm leading and the right arm following). Importantly, our ecological dynamics explanation does not disprove other, more traditional explanations of positive transfer or cross-education (e.g., Stöckel and Weigelt, 2012). Unlike the traditional explanations, however, ecological dynamics provides the tools for a formalized modeling of the intrinsic dynamics (i.e., using so-called collective variable equations of motion) (Zanone and Kelso, 1992), allowing predictions for which coordination patterns positive or negative transfer would or would not occur. Admittedly, however, we fall short of doing such formal modeling. Instead, we restricted ourselves to a more narrative description and only assessed the time taken to perform different field hockey skills, presuming that time indeed reflects the quality of coordination between the arms in handling the stick. A more formal modeling of handling the field hockey stick awaits a low-dimensional description of the involved bimanual coordination patterns.

Studies about modified equipment show that changing the equipment can have a positive effect on performance and learning (e.g., Farrow and Reid, 2010; Buszard et al., 2014; Timmerman et al., 2015; Limpens et al., 2018; Oppici et al., 2018; Brocken et al., 2020; Nor Azmi et al., 2020). At the moment, two explanations dominate in the literature. The first explanation is that modified equipment induce exploration thereby leading to new coordination patterns. In other words, modifying equipment is considered a form of task constraint manipulation that challenges the performer to adapt to the new arrangement of constraints, resulting in a larger, degenerate movement repertoire (e.g., Davids et al., 2012; Ranganathan and Newell, 2013). For instance, Brocken et al. (2020) found that practicing with a modified hockey ball that rolls less predictably than regular balls, which presumably increases movement execution redundancy and adaptivity, resulted in larger improvements in field hockey motor skills (based on a similar test as in the current study) than practicing with a regular ball. A second explanation is that equipment modification ensures better scaling of the equipment to the performer’s action capability. This leads to less conscious control and monitoring and a reduction in working memory engagement, which presumably promotes an implicit learning process (Buszard et al., 2016). For instance, Buszard et al. (2014) reported that hitting performance of children when using a scaled tennis racket was less disrupted by a cognitively demanding secondary task than with a full size equipment. With the current study, we add a third principle: equipment modification can lead to a positive transfer or cross-education between actions by utilizing the symmetry preservation principle.

### Strengths and Limitations

One of the strong points of the present study is that the practice took place during actual training. Such representative design ensures that the observed effects are genuine and practically meaningful, at least for the group of young, high-skilled children that participated. It is important however to confirm our findings with novice children and among adults across different skill levels. We expect that, in line with studies by Haaland and Hoff (2003) and Stöckel et al. (2011), similar results will be found in novice children and adults. For practitioners in other sports, it is also relevant to examine if the current cross-education effects generalize to other sports with asymmetric equipment, such as, for instance, golf. Finally, another important issue is the degree to which the observed cross-education effects depend on conscious monitoring and control and thus may increase a performer’s susceptibility to choking under pressure. It is known from research that performers who learn in an implicit way are better capable of maintain performance levels under pressure (Masters, 1992). In this study, no explicit instructions were given about the execution, but a demonstration was given. In future research it is of importance to examine whether the observed cross-education effects are learned implicitly or explicitly and thus whether a robustness against pressure is created.

In future research it is also pertinent to test the field hockey skills in more detail, instead of only taking time as variable for testing the performance of the children. In particular, it would be a critical next step to see whether the quality of coordination between the two arms and the ball has really changed by determining the (changes in) in stability of the movement coordination patterns using kinematic measures. To this end, also a formal modeling of the landscape of coordination patterns is warranted.

## Conclusion

In conclusion, the current study shows that modified equipment can be used to induce positive transfer of learning or cross-education. In practical terms, ASM REV3RSE hockey stick is very easy and directly be implemented in training, as it does not require very strict guidance or instructions for the players, or any additional education of the coaches. Also, the principle of symmetry preservation could also be explored and used for other sports that use lateralized equipment (e.g., golf), but also all other sports activities that require bilateral coordination between the limbs (e.g., basketball, judo, etc.) Additionally, the modified hockey stick enlarges variation in the training. Except for skill improvement, this may break the training routine and will keep the players “on their toes.”

## Data Availability Statement

The raw data supporting the conclusions of this article will be made available by the authors, without undue reservation.

## Ethics Statement

The studies involving human participants were reviewed and approved by the Ethical Committee of the Vrije Universiteit Amsterdam (VCWE-2018-031R1). Written informed consent to participate in this study was provided by the participants’ legal guardian/next of kin.

## Author Contributions

GS conceptualized and supervised the study. JB contributed to the analysis and data collection. JB, JK, ML, and GS contributed to the methodology and writing the original draft. JK and ML reviewed and edited the manuscript. All authors contributed to the article and approved the submitted version.

## Conflict of Interest

The author(s) declared the following potential conflicts of interest with respect to the research, authorship, and/or publication of this article: The ASM REV3RSE stick is designed by ASM. GS is one of the founders of ASM.

## Footnotes

1 https://www.reeceaustralia.com/nl/889222-8200-asm-rev3rse-hockeystick-jr/

2 https://www.athleticskillsmodel.nl/rev3rse/
