# Supplementary material for: Using Modified Equipment in Field Hockey Leads to Positive Transfer of Learning Effect
Source: Front Psychol. 2021 Apr 29;12:653004. doi: 10.3389/fpsyg.2021.653004 (PMC8116952; doi:10.3389/fpsyg.2021.653004)
Supplement: Supplementary file 1 [file Data_Sheet_1.docx]

**Appendix 1**

| **Week** | **Session** | **Type** | **Content** |
| --- | --- | --- | --- |
| Week 1 | Session 1 | Pre-test | Test |
|  | Session 2 | Exercise 1 | Dribbling and passing |
| Week 2 | Session 3 | Exercise 2 | Dribbling and controlling |
|  | Session 4 | Exercise 3 | Dribbling, controlling and passing |
| Week 3 | Session 5 | Exercise 4 | Dribbling and controlling |
|  | Session 6 | Intermediate test | Test |
| Week 4 | Session 7 | Exercise 1 | Dribbling and passing |
|  | Session 8 | Exercise 2 | Dribbling and controlling |
| Week 5 | Session 9 | Exercise 3 | Dribbling, controlling and passing |
|  | Session 10 | Exercise 4 | Dribbling and controlling |
| Week 6 | Session 11 | Post-test | Test |
|  | Training 12 | Regular training | - |
| Week 7 | Session 13 | Regular training | - |
|  | Session 14 | Retention test | Test |
