# Supplementary material for: Using Modified Equipment in Field Hockey Leads to Positive Transfer of Learning Effect
Source: Front Psychol. 2021 Apr 29;12:653004. doi: 10.3389/fpsyg.2021.653004 (PMC8116952; doi:10.3389/fpsyg.2021.653004)
Supplement: Supplementary file 2 [file Data_Sheet_2.pdf]

Appendix 2

Exercise 1 (approximately 15 minutes)

Explanation exercise:
- Player A dribbles with ball towards player B and pushes the ball.
- Player B receives the ball, dribbles the ball towards players C.
- Etc.

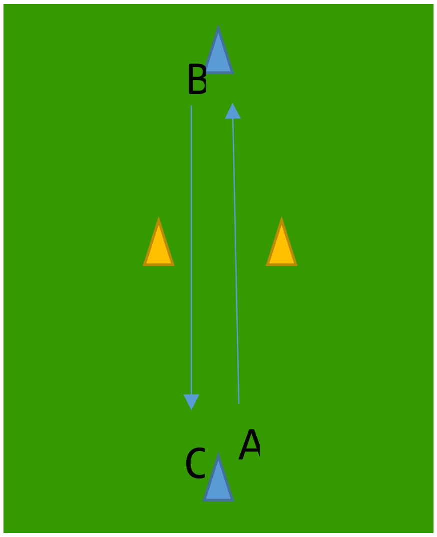

Pay attention to following focus points:
- Elbow to the outside of the body ("little bird on the elbow")
- Push while running
- Receive while in motion ("attack the ball")

Variation:
- Two extra passes
- Perform an aerial pass

Exercise 2 (approximately 20 minutes)

Explanation exercise:
- 1: Perform a broad slalom
- 2: Perform 3 aerial lifts
- 3: Perform a small slalom ("indian dribble")
- 4: Perform a regular dribble

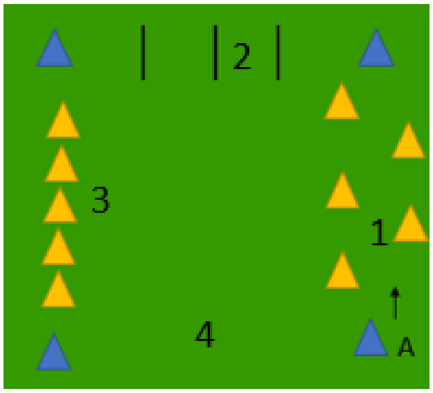

2 practice rounds of each variation, 1 relay against other team (split group in two)

Variation:
1. Normal, see explanation
2. At 1, ball around the cones, body stays in between cones; at 2, perform a "dummy" instead of the aerial lift
3. At 1 and 3, body outside of the cones, ball stays between the cones; at 2, perform a "turn".

Exercise 3 (approximately 20 minutes)

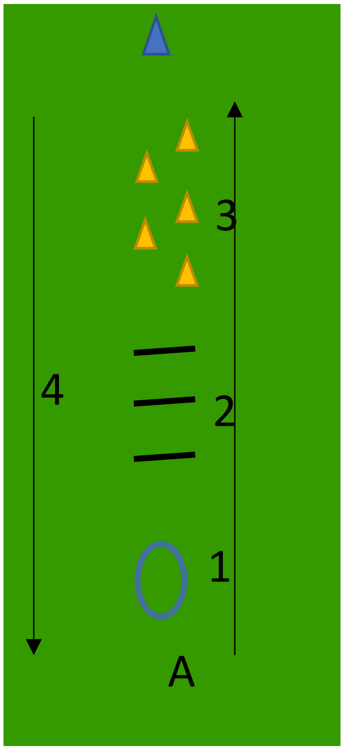

Explanation exercise:

- 1: Turn around the hoop using the forehand
- 2: Perform 3 aerial lifts
- 3: Perform a broad slalom
- 4: Dribble back to the start

1 practice rounds of each variation, 1 relay against other team (split group in three or four)

Variation:
1. Normal, see explanation
2. At 1, perform the turn using the backhand; at 2, perform a "dummy" three times instead of the aerial lift; at 3, perform the slalom with body and ball around the cones. At 4, pass the ball back to the start.
3. At 1, stand inside of the hoop and perform a turn; at 2, perform a turn three times; at 3, ball outside of the cones, body stays between the cones. At 4, perform an aerial pass back to the start.

Exercise 4 (approximately 15 minutes)

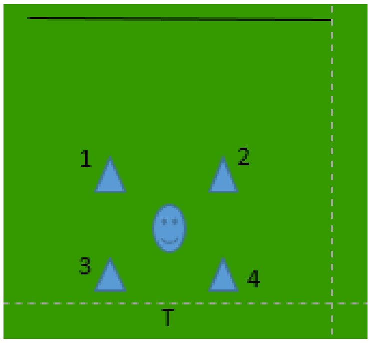

Explanation exercise:

- Players stand in a square with a ball
- Trainer calls a number, player dribbles to that cone
- At "go": players dribble as fast as they can to the line (at 20m distance)

3 rounds of each variation, each time 1 point for the winner

Variation:
1. Normal, see explanation
2. Instead of a verbally letting know what to do, do it with hand signs (players must use split vision now)
